# Integrating Real-World Data and Pharmacometrics to Bridge Evidence Gaps in Special Populations: A State-of-the-Art Review

**DOI:** 10.3390/pharmaceutics18070803

**Published:** 2026-06-29

**Authors:** Yunseok Choi, Donghyun Kim, Hyeonsu Kim, Sung Hwan Joo, Seok Jun Park, Beomjin Shin, Soyun Park, Tyler Shugg, Seungwon Yang, Won Gun Kwack, Eun Kyoung Chung

**Affiliations:** 1 Department of Pharmacy, College of Pharmacy, Kyung Hee University, Seoul 02447, Republic of Korea; cys2013@khu.ac.kr (Y.C.); waterlion3@khu.ac.kr (D.K.); lucanus2@khu.ac.kr (H.K.); shsonic95@khu.ac.kr (S.H.J.); psjqkr0824@naver.com (S.J.P.); rangers9804@khu.ac.kr (B.S.); 1223psy@khu.ac.kr (S.P.); syang345@khu.ac.kr (S.Y.); 2 Department of Regulatory Science, Graduate School, Kyung Hee University, Seoul 02447, Republic of Korea; 3 Institute of Regulatory Innovation through Science, Kyung Hee University, Seoul 02447, Republic of Korea; 4 Division of Clinical Pharmacology, Department of Medicine, Indiana University School of Medicine, Indianapolis, IN 46202, USA; tshugg@iu.edu; 5 Division of Pulmonary, Allergy and Critical Care Medicine, Kyung Hee University Hospital, Seoul 02447, Republic of Korea; 6 Institute of Integrated Pharmaceutical Sciences, Kyung Hee University, Seoul 02447, Republic of Korea; 7 Department of Pharmacy, Kyung Hee University Hospital at Gangdong, Seoul 05278, Republic of Korea

**Keywords:** pharmacometrics, real-world data, special populations, model-informed drug development, population pharmacokinetics

## Abstract

**Background/Objectives**: Special populations, including pediatric, geriatric, and organ-impaired patients, are consistently underrepresented in randomized controlled trials (RCTs), resulting in limited evidence for safe and effective dosing. Off-label use is common, and variability in drug exposure and response increases the risk of adverse drug reactions (ADRs). This review aims to examine how integrating pharmacometrics (PMX) with real-world data (RWD) can address evidence gaps by supporting dose optimization, population expansion, and safety evaluation in these vulnerable groups. **Methods**: A narrative literature review was conducted using PubMed, Embase, and Web of Science (January 2000–November 2025). Using Boolean combinations of PMX and RWD-related search terms, approximately 200–300 records were identified across the three databases; approximately 30 full-text articles were reviewed, and representative case studies were selected based on population diversity, methodological variation, and regulatory or clinical impact. **Results**: RWD–PMX integration has been applied across three domains: (i) dosing optimization through therapeutic drug monitoring (TDM)-informed PopPK modeling and model external validation in pediatric and neonatal populations; (ii) population expansion supporting dose extrapolation and regulatory decision-making for unapproved groups; and (iii) safety evaluation enabling identification of exposure–toxicity risk factors in vulnerable cohorts. **Conclusions**: Integrating PMX with RWD provides a practical and mechanistically grounded framework for evaluating dosing, treatment eligibility, and safety in populations insufficiently represented in clinical trials. Accumulating evidence indicates that RWD–PMX methodologies can complement traditional clinical research and inform regulatory decision-making. Continued refinement of data quality standards, validation practices, and guidance frameworks will be essential for broader adoption.

## 1. Introduction

Clinical trials face fundamental limitations in evaluating drug safety and efficacy across all patient groups. Strict eligibility criteria, while enhancing internal validity, inevitably exclude many vulnerable individuals. This leads to gaps in evidence for populations not adequately represented in randomized controlled trials (RCTs). An analysis of more than 5000 pediatric studies registered in ClinicalTrials.gov showed that nearly half enrolled fewer than 100 participants, underscoring the persistent challenges in generating adequately powered data for children [1]. Similar evidence gaps exist for older adults, pregnant women, and patients with renal or hepatic impairment, whose physiologic characteristics differ substantially from those of typical trial participants.

Because of these gaps, off-label prescribing has become common despite the lack of robust clinical or mechanistic support. This practice carries meaningful safety implications. In a large electronic health record (EHR)-based cohort including 46,021 adults and 151,305 prescriptions, off-label use accounted for 11.8 percent of all prescriptions, and nearly 80 percent of those uses lacked strong supporting evidence [2]. Off-label prescribing was associated with higher risk of adverse drug reactions (ADRs), particularly when scientific justification was weak, highlighting the urgent need for evidence-based dosing strategies in underrepresented populations.

In parallel with these challenges, interest has grown in the complementary roles of real-world data (RWD) and pharmacometrics (PMX). RWD, including electronic health records, registries, and therapeutic drug monitoring, capture routine clinical practice and offer enhanced external validity, especially for populations often excluded from trials. However, real-world datasets frequently contain incomplete dosing information, inconsistent laboratory measurements, and unstructured documentation that complicate causal inference and necessitate rigorous study design and data curation [3,4]. PMX, encompassing population pharmacokinetics (PopPK), physiologically based pharmacokinetic (PBPK) modeling, and pharmacokinetic/pharmacodynamic (PK/PD) analysis, provides mechanistic insights into drug disposition and exposure-response relationships. These models enable quantitative evaluation of clinical scenarios that are difficult to test prospectively. Yet models developed solely from controlled trial data may not reflect the full range of variability present in special populations, including differences in organ function, disease severity, and polypharmacy [3].

Integrating RWD with PMX approaches helps overcome the inherent limitations of each methodology. RWD introduce broader clinical representativeness and population-level diversity, while PMX contributes to structured inference and mechanistic rigor. Together, RWD–PMX methodologies provide an opportunity to generate evidence that is both clinically relevant and biologically grounded, thereby supporting improved dosing, safety evaluation, and treatment optimization for populations traditionally underrepresented in clinical research.

This review summarizes how RWD–PMX integration has been jointly applied to address critical evidence gaps in special populations. We highlight three major areas: (i) optimization of dosing strategies, (ii) the development of evidence to support treatment in populations not adequately studied in clinical trials, and (iii) the evaluation of safety outcomes in individuals at heightened risk of adverse reactions. These applications demonstrate the potential of integrated approaches to generate more robust and clinically meaningful evidence for populations historically underrepresented in drug development. While RWD–PMX integration as a general concept has received increasing attention in the clinical pharmacology literature, existing reviews have addressed this primarily from a broad drug development perspective [5,6]. What remains comparatively lacking is a dedicated synthesis organized specifically around the unique challenges faced by special populations, groups defined by developmental or physiological characteristics that fundamentally alter drug behavior and limit the applicability of conventional trial-derived evidence. This review addresses the gap by providing a population-centered framework that maps RWD–PMX methodologies to the specific evidence needs of pediatric, geriatric, and organ-impaired patients, integrating current regulatory context and identifying persistent methodological challenges that require further attention.

## 2. Literature Search and Scope

This review was conducted as a narrative synthesis of published studies examining the integration of RWD with PMX approaches in special populations. A structured search was performed in PubMed, Embase, and Web of Science for the period of January 2000 to November 2025.

Search terms were designed to capture both PMX methodologies and RWD sources. The primary Boolean search strategy combined the following term clusters: (“pharmacometrics” OR “population pharmacokinetics” OR “physiologically based pharmacokinetic” OR “PBPK” OR “PK/PD modeling”) AND (“real-world data” OR “electronic health records” OR “registries” OR “therapeutic drug monitoring”) AND (“special populations” OR “pediatric” OR “geriatric” OR “renal impairment” OR “hepatic impairment”). Reference lists of included studies were also screened to identify additional relevant publications. The initial search yielded approximately 200~300 records across the three databases. Following removal of duplicates and screening of titles and abstracts for relevance, approximately 30 full-text articles were reviewed in detail.

Studies were included if they:

(1) Applied PMX methods such as PopPK, PBPK, PK/PD, or quantitative systems pharmacology (QSP) modeling;
(2) Incorporated RWD sources such as electronic health records, registries, claims databases, or therapeutic drug monitoring;
(3) Involved special populations or generated evidence relevant to groups underrepresented in clinical trials.

Studies were excluded if they relied solely on clinical trial data without RWD, reported only observational findings without a modeling component, or focused exclusively on methodological development without clinical application.

**Figure 1:**
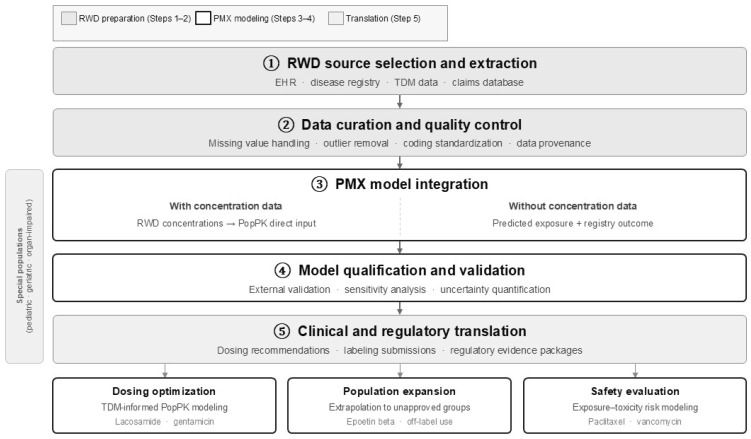
Conceptual workflow for integrating real-world data (RWD) with pharmacometric (PMX) approaches in special populations. Light gray shading (Steps 1–2) = RWD preparation phase; white fill with dark border (Steps 3–4) = PMX model integration and validation; medium gray (Step 5) = translation to clinical and regulatory outputs across three application domains. EHR, electronic health record; PopPK, population pharmacokinetics; TDM, therapeutic drug monitoring.

Because the goal of this review was to synthesize conceptual and practical insights rather than conduct a quantitative meta-analysis, no formal quality assessment tool was applied. Instead, representative case studies were selected to reflect diversity across three criteria: (i) population type (pediatric, geriatric, and organ-impaired patients); (ii) PMX approach (PopPK, PBPK, and PK/PD modeling); (iii) demonstrated regulatory or clinical impact, including labeling changes, dose guideline revisions, or reduction in confirmatory trial requirements. In practice, RWD–PMX integration proceeds through five operational steps that are common across application domains: (1) RWD source selection and extraction from EHRs, registries, or TDM repositories, with assessment of data completeness and population representativeness; (2) data curation and quality control, including missing value handling, outlier identification, standardization of clinical coding systems, and documentation of data provenance; (3) integration with PMX model structures, either by using RWD-derived drug concentrations directly as model inputs for PopPK estimation, or by linking model-predicted exposure metrics to registry-based clinical outcome data when concentration measurements are unavailable; (4) model qualification and validation, including assessment of predictive performance, sensitivity analyses, and evaluation of robustness under real-world variability; and (5) translation of modeling outputs into clinical dosing recommendations, labeling submissions, or regulatory evidence packages. This workflow is illustrated conceptually in Figure 1.

## 3. Applications of RWD–PMX in Special Populations

### 3.1. Dosing Optimization Using RWD–PMX

Optimizing dosing is one of the most mature and widely demonstrated applications of integrating RWD–PMX. Special populations often lack dedicated PK studies due to ethical or logistical constraints, and real-world drug concentration data have become key to informing PopPK models and refining dose strategies.

#### 3.1.1. RWD with Drug Concentrations

When RWD include drug concentration measurements, PopPK modeling can directly characterize exposure variability and identify covariates influencing drug disposition.

A well-described example is lacosamide use in very young children. Before formal pediatric approval, off-label use generated TDM data that enabled PopPK modeling. Younger children required higher weight-normalized doses to reach therapeutic levels, and strong inducers such as phenobarbital and felbamate substantially increased clearance. These findings, later reflected in pediatric labeling, show how RWD can supplement sparse trial data and refine assumptions about maturation and dosing in early childhood [7] (Table 1).

Gentamicin in neonates offers another example. Routine TDM produced large real-world concentration datasets that were used to externally validate existing PopPK models. A nationwide EHR cohort of more than 4500 infants confirmed overall model accuracy while revealing systematic overprediction of peak and trough levels, partly due to timing inconsistencies and assay variation in routine care. Such evaluations strengthen confidence in clinical dosing strategies and highlight where models may require refinement [8].

#### 3.1.2. RWD Without Drug Concentrations

Even when concentration data are unavailable, RWD can still support exposure–response analyses when combined with existing PopPK or PBPK models.

In older adults with status epilepticus, registry data paired with PopPK modeling of levetiracetam showed that loading doses generally achieved expected exposures but did not correlate clearly with treatment response. Variability in real-world dosing approaches, sampling times, and the drug’s short half-life limited interpretability, illustrating how RWD–PMX can challenge assumptions about dose–response relationships in acute care [9].

RWD can also inform the construction of virtual populations for PBPK simulations. In obese children, demographic data from national datasets were used to model clindamycin and sulfamethoxazole/trimethoprim dosing. Simulations aligned with existing pediatric recommendations and demonstrated that although weight-normalized clearance is reduced in obesity, current regimens with dose caps achieve therapeutic exposure. These findings show how RWD-informed virtual cohorts can help stress test dosing in high-risk pediatric subgroups [10].

### 3.2. Expansion of Target Patient Populations

RWD frequently reveal substantial off-label medication use in groups not included in clinical trials. When combined with PMX, these data help evaluate whether physiologic differences warrant adjusted dosing and whether treatment responses in unapproved populations align with model predictions.

A key example is methoxy polyethylene glycol–epoetin beta. Although approved only for adults with anemia, it is widely prescribed to children on dialysis. PopPK and PK/PD models developed from adult early-phase trials were used to simulate pediatric dosing, and predictions matched hemoglobin responses observed in the International Pediatric Dialysis Network registry. Importantly, this RWD–PMX evidence supported regulatory decisions; matching exposure–response relationships across age groups justified a major reduction in the planned pediatric confirmatory study size, from 150 patients to a 40-patient single-arm design. This case illustrates how integrated evidence can validate extrapolation, reduce trial burden, and support more equitable access to therapies [11].

Together, these examples demonstrate how RWD–PMX can transform off-label practice into evidence-supported dosing frameworks and extend therapeutic indications beyond the populations traditionally studied in clinical trials.

### 3.3. Safety Evaluation in Vulnerable Populations

Special populations experience safety risks that are not fully captured in RCTs due to differences in physiology, multimorbidity, and patterns of polypharmacy. Integrated RWD–PMX approaches allow quantitative evaluation of exposure–toxicity relationships and identification of risk factors across heterogeneous real-world cohorts.

Paclitaxel-induced myelosuppression provides a representative case. By linking EHR-derived laboratory values with a validated PK/PD model, investigators identified baseline absolute neutrophil count (ANC) as the dominant predictor of toxicity. In contrast, chronological age had no measurable effect despite widespread assumptions to the contrary. Variation in chemotherapy regimens produced larger differences in toxicity risk than age, highlighting how real-world treatment patterns introduce clinically meaningful heterogeneity absent from trial populations. Where drug concentrations were unavailable, reconstructed exposure profiles still enabled robust toxicity assessment [12] (Table 1).

These findings illustrate the value of combining mechanistic modeling with real-world clinical outcomes to refine or overturn long-standing assumptions about safety in vulnerable groups. Anticoagulant safety in elderly and renally impaired patients represents another clinically important domain. Renal function decline with aging alters the pharmacokinetics of direct oral anticoagulants (DOACs), which undergo substantial renal elimination, leading to drug accumulation and elevated bleeding risk. Integrating real-world data on renal function trajectories with PopPK models enables quantitative estimation of individual exposure profiles and supports identification of patients at highest risk of hemorrhagic events, informing individualized dose adjustment decisions that are not achievable through fixed-dose prescribing [13]. Vancomycin-associated nephrotoxicity in critically ill patients illustrates the utility of RWD–PMX in the intensive care unit (ICU) setting. ICU patients experience rapid and unpredictable changes in renal function, making standard weight-based dosing unreliable. TDM data collected during routine ICU care have been used to develop PopPK models that characterize substantial inter-individual variability in vancomycin clearance. Bayesian individualized dosing based on these models, targeting area-under-the-curve (AUC)/minimum inhibitory concentration (MIC)-guided exposure, has been shown to significantly increase attainment of therapeutic targets while reducing nephrotoxicity incidence compared with conventional trough-based approaches [14]. Despite these demonstrated applications, safety evaluation using RWD–PMX remains more methodologically challenging than efficacy-focused work. Key barriers include confounding by indication and disease severity, the low frequency of serious adverse events requiring large datasets for signal detection, time-varying exposure–toxicity relationships that complicate causal attribution, and the incomplete nature of longitudinal outcome data in routine clinical documentation. More broadly, RWD studies show that off-label prescribing itself is associated with substantially higher rates of adverse drug events, particularly when unsupported by strong evidence, underscoring the need for model-informed evaluation in such settings [2]. Given that vulnerable populations are often the very groups most likely to receive off-label therapy due to the lack of trial data, the resulting combination of physiologic sensitivity and evidence gaps substantially amplifies ADR risk. Integrating RWD–PMX is therefore essential not only for understanding toxicity mechanisms but also for mitigating the compounded harm that arises when off-label use intersects with clinical vulnerability.

### 3.4. Methodological Considerations and Limitations

Despite the demonstrated utility of RWD–PMX integration, several methodological challenges require explicit acknowledgment. Missing data represent one of the most pervasive challenges in RWD–PMX research. Real-world datasets routinely contain incomplete dosing records, unscheduled drug concentration measurements, and inconsistently documented covariates such as body weight, renal function, and concomitant medications. The choice of imputation strategy, ranging from complete case analysis to multiple imputation or model-based approaches, can substantially influence pharmacokinetic parameter estimates and their precision. Transparency in reporting missing data mechanisms and the sensitivity of conclusions to imputation assumptions is therefore essential for reproducibility [3,4].

Time-varying covariates and immortal time bias introduce additional complexity. In RWD cohorts, patient characteristics such as renal function, body weight, and comedications change over the observation period, and failure to account for their time-varying nature can distort exposure–response relationships. Immortal time bias arises when the exposure definition inadvertently creates a period of follow-up during which the outcome cannot occur, artificially inflating the apparent benefit or safety of the treatment under study. Approaches such as target trial emulation and time-varying exposure modeling have been proposed to minimize these distortions and improve internal validity [3].

A fundamental tension in RWD–PMX research is the trade-off between external and internal validity. Real-world data enhance external validity by capturing the full spectrum of patient heterogeneity encountered in clinical practice, including disease severity, polypharmacy, and organ dysfunction. However, the absence of randomization means that unmeasured confounders can bias exposure–outcome associations. The mechanistic structure of PMX models partially mitigates this limitation by incorporating physiologically grounded constraints on how covariates influence drug disposition and effect, but cannot fully compensate for fundamental deficiencies in data quality or study design [4].

The relative maturity of different PMX approaches also varies when integrated with real-world data. PopPK modeling is the most established methodology in this context, supported by extensive regulatory precedent and well-validated software platforms. PBPK modeling offers mechanistic depth by incorporating organ-specific physiological parameters, making it particularly valuable for pediatric extrapolation and organ impairment scenarios; however, it requires parameter assumptions that may not be directly estimable from RWD, necessitating careful sensitivity analysis. QSP models are the most systems-level approach but currently have the fewest documented examples of direct integration with routine real-world data sources. As the field evolves, hybrid frameworks combining PopPK-derived variability with PBPK mechanistic structure are emerging as a promising strategy for bridging these methodological gaps in the study of special populations [3].

## 4. Extended Applications Across the Drug Development Continuum

### 4.1. Target Identification and Validation

Recent advances in data integration have enabled the use of cohort-level clinical data, transcriptomic profiles, and proteomic measurements to inform QSP and other related PMX modeling frameworks. Combining these data with network-based analyses including QSP or PBPK models allows construction of mechanistic representations that better reflect human disease biology. Incorporating in vitro assay parameters further enables estimation of target-site concentrations required for engagement and prediction of downstream pathway effects or biomarker changes [15,16].

A representative example is the systems biology model developed for CD4+ T cell-mediated immune disorders, where integrated transcriptomic and proteomic datasets led to the identification of 68 potential therapeutic targets [17]. This illustrates how patient-derived biological information can support hypothesis generation and model-based target validation. Similar principles shape the “three-pillar paradigm” in drug development, which emphasizes adequate target-site exposure, target engagement, and pharmacologic activity. PMX and PBPK approaches increasingly underpin these assessments, providing a structured means to evaluate whether candidate targets are pharmacologically and clinically tractable during early development [15,18].

### 4.2. Drug–Drug Interaction Assessment

Standard clinical trials rarely reflect the diversity of concomitant medication use seen in practice, limiting their ability to fully characterize drug–drug interactions (DDI). In contrast, RWD captures complex polypharmacy patterns, particularly in older adults and patients with multimorbidity. Integrating these datasets with in vitro transporter assays and trial data has revealed clinically meaningful DDIs that may not emerge in controlled settings [19].

When combined with PMX, RWD-based DDI assessments become more informative. PMX models help refine PK/PD predictions, detect emerging interaction signals, and contextualize potential safety concerns. Recent studies using large-scale real-world datasets show that these combined methodologies can efficiently prioritize which putative interactions require prospective evaluation and which may be addressed through labeling or risk-mitigation strategies [5,6].

### 4.3. Pharmacogenomics and Model-Informed Precision Dosing

Growing adoption of genomic testing has expanded opportunities to link genetic variation with real-world treatment outcomes. Oncology, where genotyping is often integrated into diagnostic workflows, provides a clear setting for these applications.

Tamoxifen serves as a well-established example. Model-informed precision dosing that incorporates CYP2D6 phenotype, PMX modeling, and TDM data has achieved substantially higher rates of target endoxifen concentrations compared with fixed-dose or genotype-guided dosing alone [20]. These findings demonstrate that pharmacogenomic information is most effective when quantitatively embedded within exposure–response models rather than interpreted independently.

### 4.4. Pharmacoeconomic Modeling Integrated with PMX

Linking PMX outputs with pharmacoeconomic frameworks allows quantitative evaluation of potential value early in development, helping assess clinical benefit, budget impact, and cost-effectiveness before large trials are undertaken [21].

RWD enhances these analyses by providing realistic estimates of adherence, persistence, resource use, and background event rates. For instance, a pharmacoeconomic assessment of a novel xanthine oxidase inhibitor integrated PK/PD predictions of uric acid reduction into a cost-effectiveness model to compare the value of improved treatment forgiveness relative to existing therapies. This example illustrates how incorporating real-world patterns of medication use can inform strategic decisions and refine early development plans [22].

## 5. Regulatory and Policy Considerations

The integration of RWD–PMX is increasingly recognized by regulatory agencies as a complementary source of evidence, yet its broader adoption requires clearer scientific and policy frameworks. In parallel, recent U.S. regulatory initiatives have explicitly emphasized the combined use of modeling and real-world evidence. Prescription Drug User Fee Act (PDUFA) VII identified model-informed drug development (MIDD) as a priority pathway for regulatory decision support, and the FDA’s Advancing Real-World Evidence (RWE) Program Roadmap underscored the agency’s commitment to expanding the use of real-world data in both pre- and post-approval settings [23]. In addition, the 21st Century Cures Act provided the statutory foundation for incorporating RWD/RWE into regulatory evaluations [24], collectively creating a policy environment that increasingly encourages integrated RWD–PMX approaches. Although both RWD and PMX are now routinely incorporated into submissions, current guidelines from the FDA and EMA primarily address these domains separately, leaving methodological expectations for their combined use underdefined [25,26,27]. Together, these initiatives signal that integrated use of RWD and model-based approaches is shifting from optional innovation to an expected component of contemporary regulatory science. A landmark development in this regulatory evolution is the International Council for Harmonisation (ICH) M15 General Principles for Model-Informed Drug Development guidance, adopted by the ICH Assembly in January 2026 and published in the Federal Register in June 2026 following the December 2024 draft [27]. The M15 guidance establishes a harmonized MIDD assessment framework across major ICH regions, including the FDA, EMA, and PMDA. Its key provisions directly relevant to RWD–PMX integration include pre-specified MIDD plans documenting the intended use of modeling evidence; standardized model qualification criteria encompassing quantitative diagnostics and clinical interpretability; explicit recognition of real-world data as a legitimate input to MIDD submissions; and requirements for uncertainty quantification and transparent communication. The finalization of M15 represents a critical signal that RWD–PMX integration is expected to follow a harmonized, auditable framework across global submissions, a shift that will particularly benefit special populations for whom model-based extrapolation from limited data remains the primary pathway to evidence generation.

A central challenge remains in the inherent variability and incompleteness of RWD. Missing dosing records, inconsistent laboratory data, and heterogeneity in clinical documentation complicate model development and can undermine causal interpretation. Recent regulatory discussions have therefore emphasized the need for minimum data quality standards, transparent preprocessing workflows, and explicit documentation of data provenance to ensure reproducibility of RWD-derived evidence [28]. Model validation further complicates RWD–PMX integration. Traditional PMX models are typically validated using controlled clinical trial datasets, whereas RWD requires additional considerations, such as alignment of structural assumptions across heterogeneous populations and demonstration of robustness under real-world practice variability. Emerging recommendations advocate validation frameworks that balance quantitative diagnostics with clinical interpretability to support regulatory decision making [29]. Examples from recent submissions demonstrate that RWD–PMX can enable pediatric extrapolation, refine dosing in underrepresented populations, and characterize exposure–response relationships when trial data are limited [30]. However, standardized criteria defining when such evidence is sufficient for labeling changes or dose justification remain in development.

Ultimately, the regulatory utility of RWD–PMX will depend on coordinated efforts to harmonize data infrastructures, standardize modeling practices, and develop guidance explicitly addressing integrated evidence streams. As real-world datasets expand and modeling approaches mature, unified regulatory frameworks will be essential for ensuring consistent, rigorous, and ethically grounded application across drug development and post-marketing evaluation [31,32].

## 6. Conclusions

Special populations such as pediatric, geriatric, and organ-impaired patients remain persistently underrepresented in conventional clinical trials. This gap contributes to uncertainty in dosing, substantial variability in drug response, and a higher risk of adverse outcomes in real-world practice. Integrating RWD with PMX methodologies offers a pragmatic and mechanistically grounded pathway to address these limitations by generating evidence that cannot be obtained through randomized trials alone. As drug development increasingly adopts a lifecycle perspective, integrated RWD–PMX approaches align closely with the two major pillars of model-informed regulatory science: Model-Informed Drug Discovery and Development (MID3) and Model-Informed Precision Dosing (MIPD). Within the MID3 framework, combining real-world evidence with mechanistic modeling can inform early decision-making, reduce uncertainty in dose selection, and support extrapolation strategies that minimize the need for large confirmatory trials in vulnerable populations. In the clinical use stage, RWD-enabled PMX models form the basis of MIPD by capturing real-world variability in physiology, comorbidities, and treatment patterns—facilitating individualized dosing strategies that are not achievable through traditional trial designs alone.

Evidence across diverse therapeutic areas shows that combining RWD and PMX can refine dosing strategies, support extrapolation to populations not included in clinical trials, and identify clinically meaningful safety risks that may be obscured in controlled environments. These applications illustrate how this integrated approach strengthens both scientific understanding and clinical decision making.

Beyond these established uses, RWD combined with PMX now contributes to broader drug development activities such as target evaluation, drug–drug interaction assessment, pharmacogenomic-guided precision dosing, and early health economic analysis. Incorporating real-world clinical heterogeneity into mechanistic models further supports MID3 by reducing uncertainty, shortening development timelines, and enhancing the feasibility of studying vulnerable populations.

To fully realize the regulatory and clinical potential of this approach, clearer expectations for model validation, improved data quality standards, and harmonized guidance for integrated evidence generation are needed. Continued collaboration among regulatory agencies, industry, academic groups, and data-generating networks will be essential to ensure methodological rigor, transparency, and consistency.

Overall, integrating RWD with PMX offers a promising strategy for generating actionable and patient-centered evidence for populations that have historically been underrepresented in drug development. As methodological and policy frameworks continue to mature, this approach is likely to play an increasingly important role in advancing safe and effective medication use across diverse patient groups.

## Figures and Tables

**Table 1:** Summary of representative case studies illustrating real-world data–pharmacometrics (RWD–PMX) integration in special populations.

Population, Drug	RWD Source	PMX Method	Key Finding	Clinical Impact	Regulatory Impact	Ref.
***3.1*** *Dosing optimization*						
Pediatric (<4 yr), lacosamide	TDM data (off-label clinical use)	PopPK (NONMEM)	Higher weight-normalized doses required; phenobarbital and felbamate significantly increased clearance	Lacosamide dosing refined in young children; maturation-based dosing guidance established	Findings incorporated into FDA pediatric labeling	[7]
Neonates, gentamicin	Nationwide EHR cohort (n > 4500)	PopPK external validation	Overall model accuracy confirmed; systematic overprediction of peaks/troughs identified	Gentamicin dosing nomograms validated; model refinement needs identified for routine care	Supports regulatory framework for model external validation with real-world data	[8]
Older adults (status epilepticus), levetiracetam	Registry + clinical records	PopPK	Loading doses achieved expected exposures but did not correlate with treatment response	Challenged dose–response assumptions in acute seizure; highlighted needs for better pharmacodynamic endpoints	Illustrates RWD limitations for dose–response inference without controlled exposure sampling	[9]
Obese children, clindamycin and SMX/TMP	National demographic datasets	PBPK (virtual population)	Current dose-capped regimens achieve therapeutic exposure despite reduced weight-normalized clearance	Existing dosing guidelines supported for clindamycin and SMX/TMP in obese pediatric patients	RWD-informed virtual populations validated as simulation tool for regulatory submissions	[10]
***3.2*** *Expansion of target patient populations*						
Pediatric dialysis (anemia), methoxy polyethylene glycol–epoetin beta	IPDN registry (hemoglobin outcomes)	PopPK + PK/PD simulation	Pediatric exposure–response matched adult model predictions; hemoglobin responses aligned across age groups	Dose extrapolation from adults to unapproved pediatric dialysis population supported	Confirmatory study reduced from 150 to 40 patients (single-arm); regulatory acceptance of RWD–PMX evidence	[11]
***3.3*** *Safety evaluation in vulnerable populations*						
Adult cancer patients, paclitaxel	EHR-derived laboratory values (ANC, CBC)	PK/PD (myelosuppression)	Baseline ANC was the dominant toxicity predictor; chronological age had no measurable effect	Refuted age-based dose reduction assumptions; ANC identified as actionable risk stratification tool	Demonstrates RWD–PMX can overturn trial-derived safety assumptions via exposure reconstruction	[12]
Elderly NVAF patients, rivaroxaban	Hospital clinical/TDM data (rivaroxaban)	PopPK + Monte Carlo simulation	eGFR identified as major covariate; exposure (AUC) significantly associated with hemorrhagic risk	Supports individualized dose reduction for DOACs in elderly patients with renal impairment	Real-world exposure–risk quantification informing precision dosing decisions	[13]
Critically ill (ICU), vancomycin	Routine TDM data (vancomycin; meta-analysis n > 4000)	PopPK + Bayesian dosing	AUC-guided Bayesian dosing significantly reduced nephrotoxicity vs. trough-based monitoring	Bayesian AUC/MIC-targeted dosing recommended; reduces adverse drug events in patients with unstable renal function	Supports regulatory submissions for precision dosing tools in critically ill patients	[14]

Abbreviations: ADR, adverse drug reaction; ANC, absolute neutrophil count; AUC, area under the concentration–time curve; CBC, complete blood count; DOAC, direct oral anticoagulant; eGFR, estimated glomerular filtration rate; EHR, electronic health record; ICU, intensive care unit; IPDN, International Pediatric Dialysis Network; MIC, minimum inhibitory concentration; NVAF, nonvalvular atrial fibrillation; PBPK, physiologically based pharmacokinetic; PK/PD, pharmacokinetic/pharmacodynamic; PopPK, population pharmacokinetics; RWD, real-world data; SMX/TMP, sulfamethoxazole/trimethoprim; TDM, therapeutic drug monitoring.

## Data Availability

All data generated or analyzed during this study are included in this published article. Additional datasets used during the narrative review are available from the corresponding authors upon reasonable request.

## Abbreviations

The following abbreviations are used in this manuscript:

ADR	Adverse Drug Reaction
ANC	Absolute Neutrophil Count
DDI	Drug–Drug Interaction
EHR	Electronic Health Record
EMA	European Medicines Agency
FDA	U.S. Food and Drug Administration
ICH	International Council for Harmonisation
ICU	Intensive Care Unit
MIDD	Model-Informed Drug Development
MID3	Model-Informed Drug Discovery and Development
MIPD	Model-Informed Precision Dosing
PBPK	Physiologically Based Pharmacokinetic
PD	Pharmacodynamic
PDUFA	Prescription Drug User Fee Act,
PK	Pharmacokinetic
PMX	Pharmacometrics
PopPK	Population Pharmacokinetics
QSP	Quantitative Systems Pharmacology
RCT	Randomized Controlled Trial
RWD	Real-World Data
RWE	Real-World Evidence
TDM	Therapeutic Drug Monitoring
